# Evaluation of the parameters affecting the loading of anticancer drug Paclitaxel on coated gold nanoparticles for breast cancer treatment

**DOI:** 10.1049/nbt2.12121

**Published:** 2023-02-27

**Authors:** Afrooz kharazmi, Neda Attaran

**Affiliations:** ^1^ Department of Biomedical Engineering Applied Biophotonics Research Center Tehran Science and Research Branch Islamic Azad University Tehran Iran; ^2^ Department of Medical Nanotechnology Applied Biophotonics Research Center Tehran Science and Research Branch Islamic Azad University Tehran Iran

**Keywords:** cancer, nanomedicine, nanoparticles

## Abstract

The purpose of this study is the design and synthesis of gold nanoparticles (GNPs) conjugated with paclitaxel and to investigate the parameters affecting the stability of synthesised nanoparticles with drug delivery capability. Here, synthesised GNPs were coated with polyethylene glycol. Then these particles were conjugated with paclitaxel under different conditions and the physical and structural characteristics, as well as the factors affecting the loading of paclitaxel on nanoparticles, were evaluated by ultraviolet spectrophotometer, fourier transform infrared spectroscopy, transmission electron microscopy, dynamic light scattering and zeta potential apparatus. It was found that pegylated GNPs have a limited loading capacity at the time of 24 h of incubation and the Paclitaxel loading was observed to be pH dependent. The use of these particles in the treatment of breast cancer (MCF7) was also investigated using the MTT test. It was determined that the survival percentage of MCF7 cells in the presence of paclitaxel‐bound nanoparticles decreases to about 55% at the maximum measured concentration (690 μM).

## Graphical abstract

The purpose of this study is the design and synthesis of gold nanoparticles conjugated with paclitaxel and to investigate the parameters affecting the stability of synthesised nanoparticles with drug delivery capability.

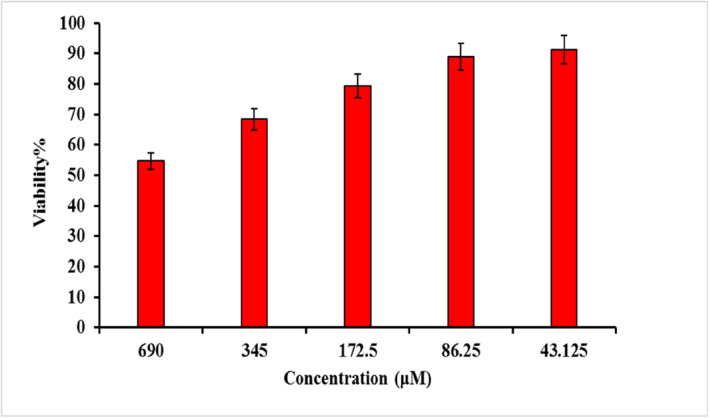

## 1 INTRODUCTION

Despite notable progress in the field of early diagnosis, timely treatment, and reduction of mortality for breast cancer in women, this type of cancer remains the most prevalent among women in many countries [1]. Breast cancer develops from breast tissue and occurs when cells in the breast mutate and grow uncontrollably, forming a tumour. Similar to other forms of cancer, breast cancer can infiltrate and expand in the surrounding tissue and can also spread to other parts of the body, creating new tumours. This process is known as metastasis [2, 3, 4, 5], which is the primary cause of cancer‐related deaths. There are various treatment options available for patients with breast cancer, including standard and commonly used methods, as well as treatments currently being tested in clinical research. One notable anticancer agent is Paclitaxel (Taxol), which has a complex structure and poor solubility in water. It is often derived from plants and is a widely used treatment for various types of cancer, particularly breast and ovarian. However, the insolubility of this drug in water and the use of toxic solvents can lead to significant side effects [6, 7]. As such, it is crucial to thoroughly research the biosynthesis mechanism and develop effective drug delivery methods to minimise these side effects.

Recent advancements in nanoscience have led to the application of nanomaterials in cancer treatment [8, 9]. Nanoparticles possess unique transport, biological, optical, magnetic, electronic, and thermal properties, which can be exploited for therapeutic purposes. The previous studies demonstrate different types of nanocarriers, such as horse spleen apoferritin [10], human serum albumin conjugated to cationic solid lipid nanoparticles [11], and solid lipid nanoparticles modified with folic acid [12] and hyaluronic acid‐chitosan‐lipoic acid nanoparticles [13], for the targeted delivery of chemotherapy drugs to treat breast cancer. Each carrier has its own advantages, such as pH‐responsive release, high entrapment efficiency, low toxicity, and specific targeting of cancer cells. The use of these nanocarriers can potentially increase the effectiveness of chemotherapy drugs while minimising side effects on normal cells. Among nanoparticles, gold nanoparticles (GNPs) have significant advantages, owing to their ease of synthesis, non‐toxicity, absorption properties, and adjustable dispersion [14, 15, 16]. Their strong dispersion and absorption properties have enabled their use in imaging cancer cells and in treatment [15, 17]. The quantum physical properties of GNPs have a high potential for improving and increasing their use in cancer diagnostic and treatment methods, as well as targeted drug delivery [16, 18, 19]. However, their use in medical applications raises some important considerations, which are as follows: a) It is important to understand the biodistribution of GNPs in order to predict their potential therapeutic effects and also to minimise any potential toxicity. Biodistribution refers to the localisation and distribution of GNPs in different organs and tissues of the body after administration. Studies have shown that AuNPs can accumulate in organs such as the liver and spleen and can also cross the blood‐brain barrier [20]. b) The presence of blood circulation time of GNPs is an important factor to consider in their use in medical applications. It refers to the time required for GNPs to be cleared from the bloodstream after administration. Longer blood circulation times may result in increased accumulation in organs and increased toxicity. Studies have shown that the circulation time of GNPs can be increased by modifying their surface with certain biomolecules such as polyethylene glycol (PEG) [21]. c) The toxicity of GNPs is a major concern in their use in medical applications. Toxicity can occur at the level of organs or at the level of genes and cells. Studies have shown that upon entering the human bloodstream, GNPs can interact with cell membranes and proteins [3, 22] and induce various biological effects such as cell membrane disruption, oxidative stress and inflammation in organs such as the liver and kidneys and can also affect the expression of certain genes [23]. Also, GNPs can cause apoptosis, inflammation, and autophagy, which may ultimately lead to cytotoxicity.

In the case of GNPs, the size and shape of the nanoparticles are important factors that affect their biodistribution, blood circulation time and toxicity [24, 25, 26]. Gold nanoparticles tend to accumulate in cells, particularly macrophages, throughout the body. The size of the nanoparticles plays a crucial role in the activation of the immune system and excretion through the kidneys, thus affecting the circulation time of nanoparticles in the body. Additionally, the surface chemistry of the nanoparticles, also plays a role in the biodistribution and toxicity of the nanoparticles. The functionalisation of GNPs can decrease protein binding in blood, evade recognition by the reticular endothelial system (RES) and reduce toxicity [27, 28, 29].

The type of functionalisation agent and compound used vary depending on the intended application. The method of functionalisation is dependent on factors such as particle size, shape, surface chemistry and structure, and material [30]. It also depends on the nature of the surface ligands, their functional groups, and the type of biomolecules that are used. Surface modification of nanoparticles can occur during the synthesis process or upon environmental exposure. For instance, charged ligands such as cetyltrimethylammonium bromide and acid citrate are commonly used as protective agents in the synthesis of nanoparticles [19, 31]. Surface decoration can alter the physical and chemical properties of nanoparticles and in turn, affect their biological effects. For example, decoration with positively charged and hydrophobic ligands on nanoparticles can increase cellular uptake, while decoration with PEG can decrease protein binding in blood and evade recognition by the RES [31, 32, 33]. Additionally, surface chemistry is a crucial factor that can contribute to adverse effects in biosystems.

The current studies are centred on creating and introducing a new category of GNPs that can transport paclitaxel, a substance with anticancer properties. Researchers are also exploring aspects that influence paclitaxel loading and identifying the ideal circumstances for utilising these nanoparticles in cancer treatment and drug delivery within the medical field. [34, 35, 36]. The development of alternative paclitaxel delivery systems, such as Cremophor® and reducing its systemic clearance, has the potential to significantly improve cancer treatment with paclitaxel. While various approaches have shown promise in replacing the Cremophor®‐based vehicle for the delivery of paclitaxel, the final product for human use is still under development [6, 37, 38]. For local targeting, PCL‐based paclitaxel surgical pastes have a high likelihood of reaching the clinic soon. Liposomal formulations of paclitaxel is another approach with great potential for systemic treatment [39, 40]. Current limitations such as limited paclitaxel delivery and clearance can be overcome by improving encapsulation efficiency and developing longer circulating liposomes.

In this study, GNPs were synthesised using the Turkevich method, where sodium citrate was used as a reducing agent. The surface of the nanoparticles was then functionalised with PEG, and finally, these nanoparticles were conjugated with paclitaxel at various concentrations and under different conditions. The structural features and factors affecting paclitaxel loading were evaluated, and the toxicity of these particles in the treatment of breast cancer cells was also examined.

## 2 MATERIALS AND METHODS

### 2.1 Materials and equipments

The chemical compounds used in this research include paclitaxel (Ectro Middle East Pharmaceutical Company), PEG (H(OCH_2_CH_2_)_n_OH, average mol wt. 8000), hydrogen tetrachloroaurate (III) trihydrate (HAuCl_4_.3H_2_O, 99.5% purity), and sodium citrate dihydrate (C_6_H_5_Na_3_O_7_.2H_2_O) were from Merck, Germany, and Fluka, Switzerland. Experiments were performed on the breast cancer MCF7 cell line obtained from the Pasteur Institute of Iran. Cell culture medium (Roswell Park Memorial Institute [RPMI]‐1640) was purchased from Grand Island Biological Company (Invitrogen, Germany). Trypsin‐ethylene diamine tetraacetic acid (EDTA), dimethyl sulfoxide (DMSO), 3‐(4,5‐dimethylthiazol‐2‐yl)‐2,5‐diphenyltetrazolium bromide (MTT) and penicillin‐streptomycin solution were purchased from Sigma‐Aldrich Corp.

The optical absorption spectrum of samples in different concentrations using a visible‐ultraviolet light spectrophotometer manufactured by the company HACH, USA (model DR6000) was determined at wavelengths of 200–800 nm. Transmission electron microscopy (TEM) using a Zeiss EM 900 from Germany was used to investigate the morphology and size of nanoparticles. DLS dynamic light scattering device (DLS, ZS‐90) from Malvern Instruments, UK, measured the particle size distribution and hydrodynamic diameter of the particles and the electric charge of particles was measured using a zeta potential measuring device (Malvern Zetasizer Nano ZS‐90) also from Malvern Instruments, UK. The fourier transform infrared (FTIR) spectra of the samples were obtained on a Thermo Nicolet Avatar 370 FTIR spectrometer from Thermo Fisher Scientific, USA.

## 3 METHODS

### 3.1 Synthesis of gold nanoparticles

In this project, according to the Anshup et al method reported, GNPs were synthesised by citrate reduction [41]. In this method, 0.01 g of HAuCl4 gold salt was added to 5 mL of deionised water. The obtained gold salt solution was added to 90 mL of water in a round bottom flask and placed on a magnetic stirrer to boil. After boiling, sodium citrate was added rapidly. By adding sodium citrate, the colour of the solution changed from pale yellow to purple and with time to dark red. After reaching the red colour, it was refluxed for another 15 min and allowed to cool at room temperature.

### 3.2 Coating gold nanoparticles with polyethylene glycol

To stabilise the synthesised GNPs, 0.1 mL of a 3% solution by weight of PEG was added to 10 mL of an aqueous solution of GNPs and placed on a magnetic stirrer for 4 h at room temperature. The presence of PEG molecules prevents the settling of nanoparticles by stabilising the solution of GNPs. Therefore, in this study, PEG was used as a coating agent on the surface of GNPs to stabilise the distribution of nanoparticles and target cells more effectively [42, 43].

### 3.3 Loading of paclitaxel on gold nanoparticles

At this stage, paclitaxel at concentrations of 345, 690 and 1380 μM was added to the solution of GNPs coated with PEG and uncoated GNPs. The solutions were placed on a magnetic stirrer for 24 h and finally centrifuged at a speed of 1500 rpm for 15 min. After examining the results of the absorption spectrum of the supernatant from the spectrophotometer, the concentration of 690 μM was considered the selected concentration of paclitaxel. Then, in the next phase, to check the time factor, Paclitaxel with the concentration of 690 μM was added to the solution of GNPs coated with PEG and placed on a magnetic stirrer for 0, 12 and 48 h. Also to check the effect of the pH environment, the pH of the solution was changed by adding HCl and NaOH aqueous solution.

## 4 MEASUREMENT

In this study, various assays and measurements were carried out to investigate the parameters affecting paclitaxel loading on the synthesised nanoparticles, which are mentioned below.

### 4.1 Optical studies of paclitaxel and the prepared nanostructures

To investigate the optical behaviour of the synthesised nanostructures and paclitaxel, a Ultraviolet‐visible (UV‐Vis) spectrophotometer was used. In this way, different concentrations of the solution containing each were prepared and its absorption spectrum was determined in the range of 200–800 nm.

The effect of coating GNPs with PEG and the loading rate of paclitaxel on nanoparticles was investigated by ultraviolet spectroscopic measurement of the centrifuged solutions of all prepared nanostructures in different conditions.

### 4.2 Morphology, size and surface studies of the prepared nanostructures

The physical properties of GNPs are strongly affected by their size because the electronic structure of gold particles changes with size and shape. The chemical properties of GNPs and their catalytic activity also strongly depend on their size and shape. Transmission electron microscope (TEM) was used to analyse the structural features and particle diameter of GNP, PEGylated GNPs (GNP‐PEG) and Paclitaxel conjugated PEGylated GNPs (GNP‐PEG‐Paclitaxel) nanostructures.

Dynamic light scattering is a physical method used to determine the distribution of particles in solutions and suspensions. This non‐destructive and fast method is used to determine the size of particles in the range of several nanometres to microns. In recent technologies, particles with a diameter of less than nanometres can also be measured with this method. This method depends on the interaction of light with the particle. In this analysis, GNP, GNP‐PEG and GNP‐PEG‐Paclitaxel solutions were used to analyse the particle size.

Zeta potential is very important to understand the properties of colloidal suspensions. It can be used as a tool to study the potential distribution in the interface in detail. This investigation can be done in the presence of simple ions and more complex systems such as surfactants, multivalent ions, polymers and even proteins. Also, the zeta potential of the sample is used to determine the tendency of the particles in the liquid to connect to each other. When charged particles are suspended in a liquid, oppositely charged ions are attracted to the suspended particles. That is, the sample with a negative charge attracts positive ions from the liquid and on the contrary, the sample with a positive charge attracts negative ions from the liquid. A good suspension does not clump and keeps its shape for a long time. In the case of fine colloids, this can be achieved by adding a suspending species to increase the zeta potential and produce the greatest amount of interparticle repulsion. In this analysis, GNP, GNP‐PEG and GNP‐PEG‐Paclitaxel solutions were used to check the electric charge of the particles.

Infrared spectroscopy is one of the most widely used spectroscopy methods because it is very effective in determining the structure of compounds. Chemical compounds have different properties due to the presence of different functional groups, and these properties can be determined with the help of infrared spectroscopy. Infrared spectra provide us with a lot of information about the structure of the analysed compounds.

### 4.3 Cell culture and cancer cell survival rate

MCF7 cells were seeded in each well of a 96‐well plate and the plate was placed in a CO_2_ incubator for 12 h. Then MCF7 cells were incubated with nanoparticles with different concentrations of 43.125, 86.25, 172.5, 345 and 690 μM for 4 h and then washed twice with phosphate buffered saline (PBS). After one day, an MTT assay was performed. 100 μL of MTT solution (0.5 mg of MTT powder in 1 cc of PBS) was added to each well of a 96‐well plate and placed in an incubator for 4 h. Finally, the MTT solution was removed from the wells and 100 μL of DMSO was added to each well. The plate was incubated for 15–20 min and read by an ELISA reader at 570 nm wavelength. Based on the optical density (OD) reading by the device, the cell survival rate was calculated. The rate of cell survival is obtained from the following relationship:

Cellviability(%)=[(ODsample–ODmedium)/(ODcontrol–ODmedium)]×100

All tests were performed in triplicate and all data are expressed as mean values ± SD (standard deviation). For statistical analysis, one‐way analysis of variance and Tukey's test as the posthoc were performed analysis using SPSS version 16 software. The value of *p* < 0.05 was statistically significant.

## 5 RESULTS AND DISCUSSION

### 5.1 Characteristics of synthesised nanostructures

**FIGURE 1:**
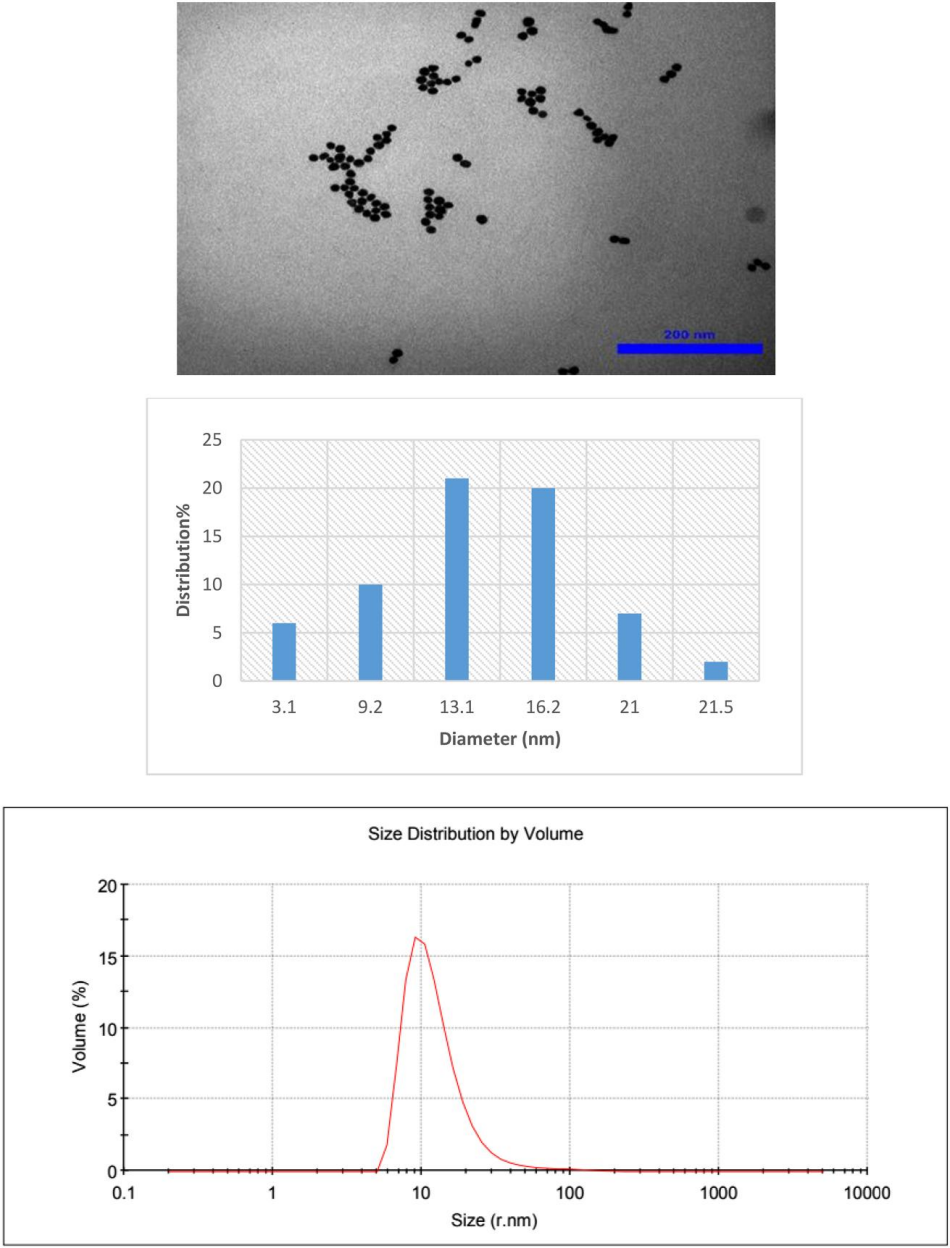
Transmission electron microscopy (TEM) images of gold nanoparticles (GNPs) with their size distribution and DLS profile.

**FIGURE 2:**
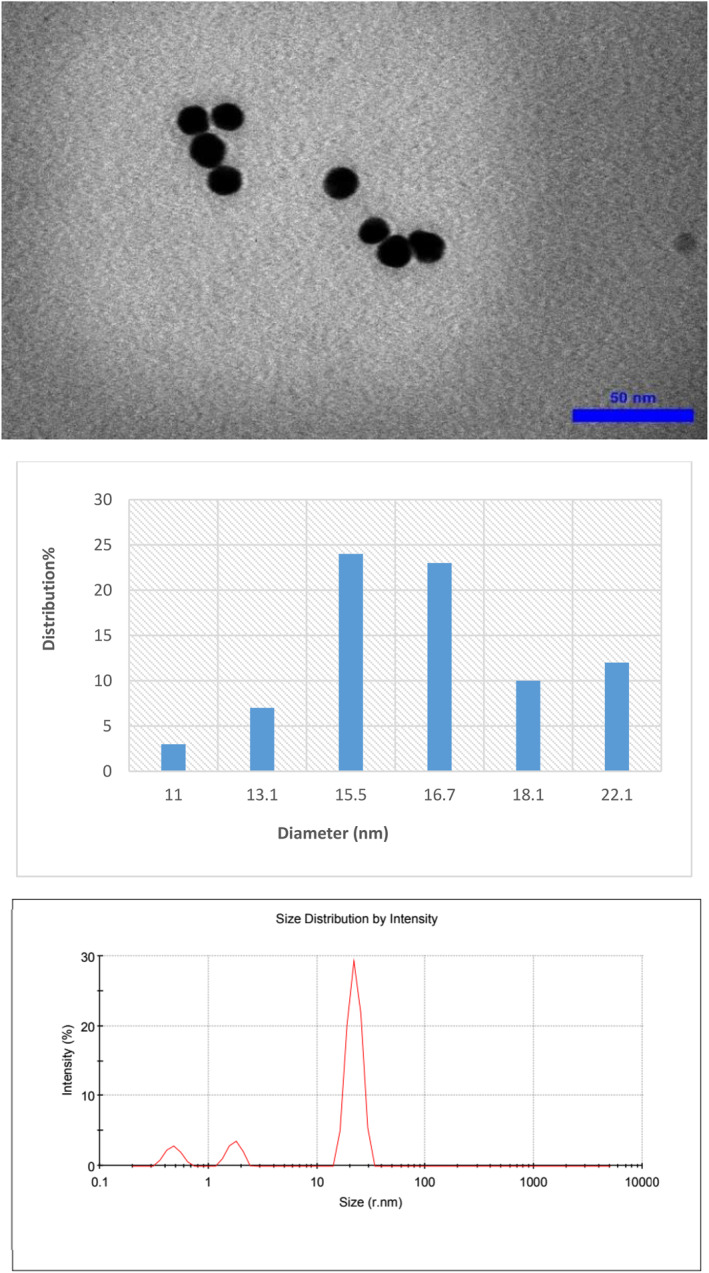
Transmission electron microscopy (TEM) images of PEG‐GNPs with their size distribution and DLS profile.

**FIGURE 3:**
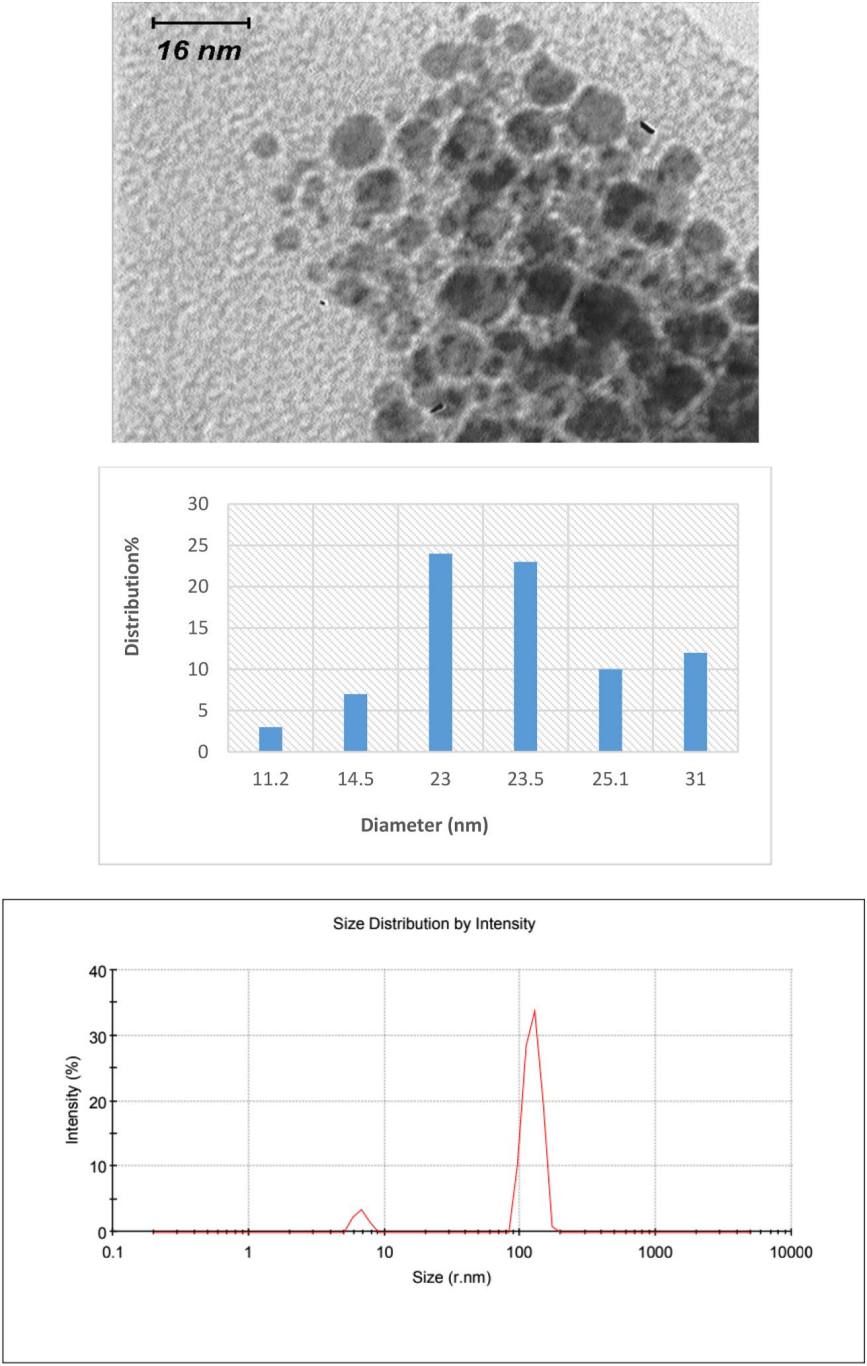
Transmission electron microscopy (TEM) images of GNP‐PEG‐Paclitaxel with their size distribution and DLS profile.

The morphology of the synthesised nanoparticles was studied by TEM. The size histogram of the nanoparticles was obtained by ImageJ software. As shown in Figures 1, 2, 3, GNP, GNP‐PEG and GNP‐PEG‐Paclitaxel have spherical shapes with mean diameters of ∼13 nm, ∼16 nm, and ∼24 nm, respectively. The size distribution of the nanoparticles was confirmed by DLS analysis. It is determined that PEG‐GNPs (22.26 nm) and GNP‐PEG‐Paclitaxel (123.6 nm) are larger than GNPs (13.56 nm) by about 9 and 109 nm, respectively. There can be several reasons why the size of the GNP‐PEG‐Paclitaxel nanoparticles as determined by TEM and DLS may differ [25, 44]. TEM images provide a high resolution image of the nanoparticles; however, the size measurement determined from TEM images are based on the projection of the particle on the image plane and it may not represent the true size of the particle. DLS measurements, on the other hand, are based on the measurement of the scattered light intensity, which is dependent on the size and refractive index of the particle. DLS measurements are sensitive to the size of the particles in solution but they are not always able to differentiate between particles with similar sizes or shapes. The discrepancy in size measurements can also be due to the fact that the TEM and DLS measurements may have been performed under different conditions, such as different buffer solutions, pH levels, and temperatures, which can affect the size and shape of the nanoparticles. Also, the surface chemistry of the nanoparticles can affect the size measurements by TEM and DLS. PEGylation on the surface of the nanoparticles, for example, can change the behaviour of the particles in solution, and this may lead to different size measurements. It is worth noting that it is common to have discrepancy between the size measurements using different techniques, and it is important to consider the size measurements obtained by TEM and DLS as complementary information. Measurements of zeta potential were utilised to verify the successful outcome of the reaction. The surface potential of PEG‐GNPs (−6.13 mV) has a more negative zeta potential than GNPs (−4.82 mV). This finding is since the OH groups in PEG induce a negative charge on the surface of nanoparticles. Also, GNP‐PEG‐Paclitaxel (−8.96 mV) is more negative than PEG‐GNPs due to the presence of OH and NH groups of paclitaxel. However, nanoparticles with a zeta potential between −10 and +10 mV are considered to be approximately neutral in charge. This means that the electrical charge on the surface of the nanoparticles is not significant enough to affect their properties or performance. This is because a zeta potential of −10 to +10 mV is considered a small range and does not create enough of an electrical force to significantly alter the behaviour of the nanoparticles. Therefore, changes in the surface charge of these nanoparticles do not have a significant impact on their performance. On the other hand, the conductivity coefficient in nanoparticles coated with polymer has a significant drop in the conductivity of nanoparticles (0.0562 S/m vs. 0.186 S/m). But again with the presence of the drug Paclitaxel, it has increased significantly (0.470 S/m), which can be attributed to the presence of aromatic rings that have electron conjugate pairs, and as a result, it has increased electron conductivity.

**FIGURE 4:**
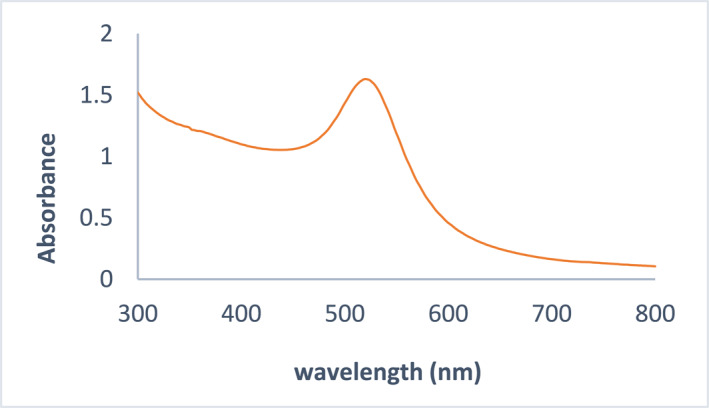
The absorption spectrum of the synthesised gold nanoparticles (GNPs).

In Figure 4, as it is clear, the absorption spectrum of GNPs at 520 nm confirms that the synthesised GNPs used in the next steps are spherical (spherical nanoparticles have a specific absorption peak in the spectral region of 520–550 nm).

**FIGURE 5:**
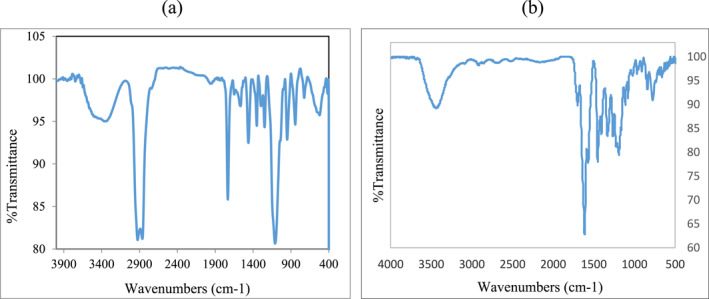
FT‐IR Spectra of (a) pegylated gold nanoparticles (GNPs) containing paclitaxel and (b) gold nanoparticles. FT‐IR, fourier transform infrared.

In order to analyse the surface of the nanostructures, FTIR spectrum was taken from the dried precipitates of pegylated GNPs containing paclitaxel (Figure 5). Meanwhile, the presence of a peak at 2883 cm^−1^ is related to aliphatic CH stretching vibrations. Also, the peak related to the carbonyl groups of paclitaxel appears at 1739 cm^−1^. Therefore, FTIR spectroscopy confirms the presence of paclitaxel and PEG on the surface of GNPs.

### 5.2 Polyethylene glycol on paclitaxel loading on gold nanoparticles

**FIGURE 6:**
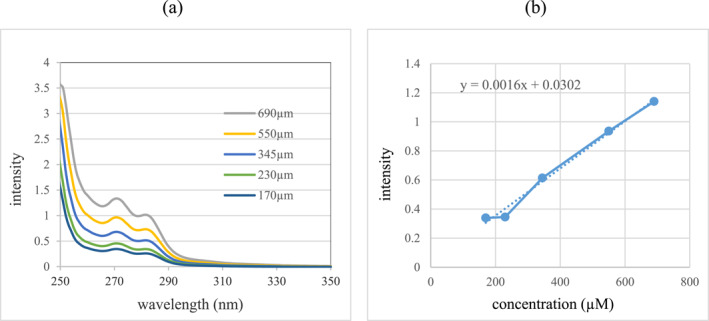
(a) Absorption spectrum curve of paclitaxel at different concentrations and (b) standard curve of paclitaxel absorption spectrum for absorption peak of 271 nm.

To investigate the loading rate of paclitaxel molecule on the synthesised GNPs and also to study the effect of the presence of PEG, first the absorption spectrum of paclitaxel in different concentrations was obtained (Figure 6). In this diagram, the absorption spectrum of the selected concentrations of 170, 230, 345, 550, and 690 μM is drawn and as can be seen, the absorption peak of paclitaxel occurs at the wavelength of 271 nm. To determine the amount of drug loading and release from the nanostructure, the standard curves that express the changes in light absorption intensity (abs), according to Paclitaxel concentration (P), were used.

The changes between the optical absorption intensity with the concentration of paclitaxel at the absorption peak of 271 nm follow the below linear equation:

$${Abs}_{271}=0.0016P+0.0302$$

In order to study the effect of the presence of PEG on the amount of paclitaxel molecule loading on GNPs, Spectrophotometric analysis technique was used. In this process, paclitaxel drug was prepared in different concentrations and the effective loading rate of GNPs and pegylated GNPs was investigated. Incubation of paclitaxel on nanoparticles was in ambient conditions for 24 h.

**FIGURE 7:**
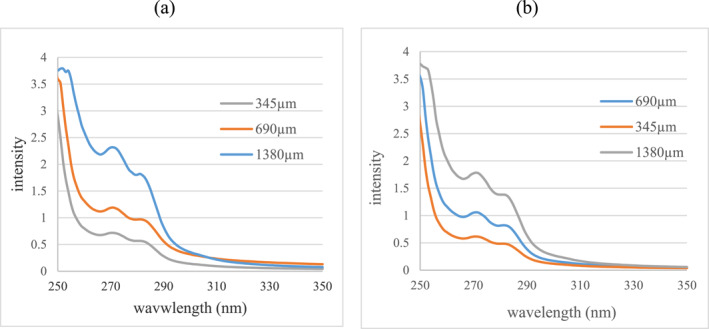
Comparison diagram of paclitaxel absorption spectrum changes in centrifuged solutions containing free paclitaxel of samples (a) GNP‐paclitaxel and (b) GNP‐PEG‐Paclitaxel at different concentrations.

Concentrations of paclitaxel used for loading study on PEG‐GNPs and GNPs were 1380, 345, and 690 μM. The amount of changes in the absorption spectrum of the centrifuged solution containing free paclitaxel at different concentrations is observed in Figure 7. It should be noted that in all stages, the concentration of GNPs was constant and 0.1 μM. In the tested concentrations, it was found that paclitaxel loading on pegylated GNPs was better than on GNPs. In other words, PEG has a more effective role in the loading process of paclitaxel.

According to the initial concentration of paclitaxel and the light absorption intensity of the centrifuged sample solutions, it is determined that the Paclitaxel concentration of 690 μM in both samples (GNP‐paclitaxel and GNP‐PEG‐Paclitaxel) was the best concentration in the process of loading on nanoparticles. While in the Paclitaxel concentration of 1380 μM did not differ significantly with the concentration of 690 μM. Also, at a concentration of 345 μM, it has the lowest loading on nanoparticles and pegylated nanoparticles. Therefore, in this study, it was found that GNPs have a limited loading capacity and it is not possible to use any desired Paclitaxel concentration for loading on the nanostructure. In Table 1, the amount of effective loading of GNPs (GNP‐paclitaxel) and pegylated GNPs (GNP‐PEG‐Paclitaxel) has been shown.

**TABLE 1:** The effective paclitaxel loading rate on the nanostructure based on the absorption spectrum of the samples.

Loading efficiency of paclitaxel (%)	Free paclitaxel concentration (μM)	Loaded paclitaxel concentration (μM)	Initial paclitaxel concentration (μM)	Nanostructure
58.52%	143.083	201.917	345	GNP‐paclitaxel
**65.46%**	238.29	451.71	690	
65.77%	472.25	907.75	1380	
66.98%	113.916	231.084	345	GNP‐PEG‐Paclitaxel
**70.08%**	206.416	483.584	690	
73.73%	362.458	1017.542	1380	

*Note*: The most effective paclitaxel loading rate is bold.

### 5.3 The effect of incubation time on the loading efficiency of paclitaxel on pegylated gold nanoparticles

**FIGURE 8:**
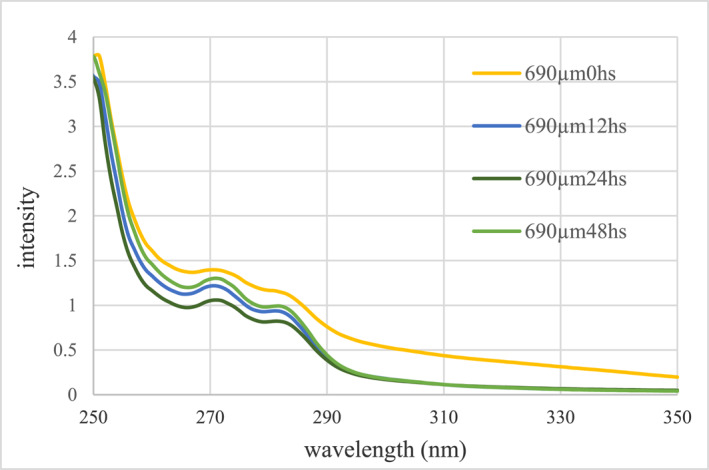
Comparison of changes in paclitaxel absorption spectrum in centrifuged solutions containing free paclitaxel at different incubation times.

In this study, samples with a concentration of 690 μM paclitaxel were prepared. Then, in order to study the effect of incubation time, the samples were incubated with pegylated nanoparticles at room temperature at zero, 12, 24, and 48 h. To investigate the effect of time on the behaviour of nanostructures and paclitaxel, an absorption spectrophotometer was used (Figure 8).

According to the initial concentration of paclitaxel and the light absorption intensity of the tested samples, it is clear that the loading of paclitaxel on the pegylated GNPs at the incubation time of 0 h at the concentration of 690 μM paclitaxel has the lowest amount of loading. But increasing the incubation time to 12 and 24 h, the loading efficiency has increased more, which is probably due to the effect of increasing the incubation time on the better placement of paclitaxel. Also, according to the graph, after 48 h, the amount of loading decreases, which shows that the long incubation time has caused the separation of the drug from the surface of the nanoparticles. Therefore, it is clear that the loading capacity has reached its maximum at the incubation time of 24 h. In Table 2, the effective loading rate of pegylated GNPs (GNP‐PEG‐Paclitaxel) at different times is shown.

**TABLE 2:** The effective paclitaxel loading rate on the nanostructure at different incubation times based on the absorption spectrum of the samples.

Loading efficiency of paclitaxel (%)	Free paclitaxel concentration (μM)	Loaded paclitaxel concentration (μM)	Initial paclitaxel concentration (μM)	Nanostructure
23.200%	529.916	160.084	0	GNP‐PEG‐Paclitaxel (concentration of 690 μM paclitaxel)
64.16%	247.25	442.75	12	
**68.93%**	214.333	475.667	24	
58.76%	284.541	405.459	48	

*Note*: The most effective paclitaxel loading rate is bold.

### 5.4 The pH effect on the loading efficiency of paclitaxel on pegylated gold nanoparticles

**FIGURE 9:**
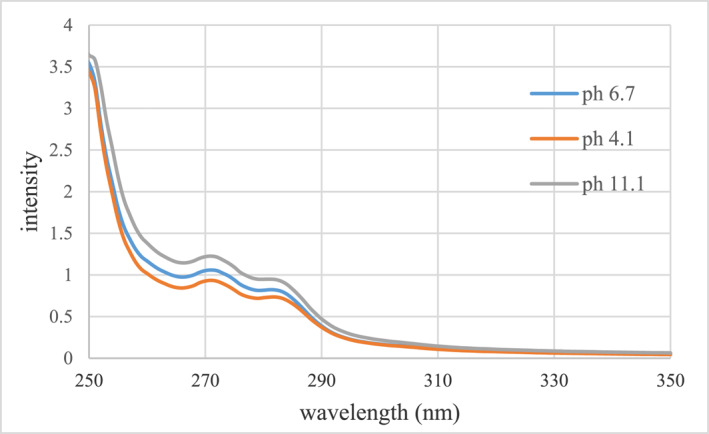
Comparison of changes in paclitaxel absorption spectrum in centrifuged solutions containing free paclitaxel at different pHs.

To study the pH effect, the reaction was carried out in two acidic and alkaline environments. The initial pH of nanoparticles was 6.7. To create an acidic environment, some acidic solution of HCl was added to the nanostructures (pH = 4.1). Also, to create an alkaline environment, some NaOH solution was added to the nanostructures (pH = 11.1). Then, the pH effect on the loading process was studied using an absorption spectrophotometer (Figure 9).

According to the initial concentration of paclitaxel and the absorption intensity of the tested samples, it is clear that the loading of paclitaxel on the pegylated GNPs has decreased in the alkaline environment at the concentration of 690 μM paclitaxel. This is probably due to the weakening of the bonds between the paclitaxel molecule and the pegylated surface of the nanoparticles in the alkaline environment. While in an acidic environment, drug loading has increased, which is probably due to the presence of acidic hydrogen in the environment and improved molecular interaction between paclitaxel and PEG. In Table 3, the effective loading rate of pegylated GNPs (GNP‐PEG‐Paclitaxel) at different pHs are shown.

**TABLE 3:** The effective paclitaxel loading rate on the nanostructure at different pHs based on the absorption spectrum of the samples.

Loading efficiency of paclitaxel (%)	Free paclitaxel concentration (μM)	Loaded paclitaxel concentration (μM)	Initial paclitaxel concentration (μM)	Nanostructure
64.25%	246.625	443.325	11.1	GNP‐PEG‐Paclitaxel (concentration of 690 μM paclitaxel)
69.17%	212.666	477.334	6.7	
**72.98%**	186.416	503.584	4.1	

*Note*: The most effective paclitaxel loading rate is bold.

### 5.5 The viability percentage of the breast cancer cells treated with GNP‐PEG‐Paclitaxel

MCF7 is a breast cancer cell line obtained from the Pasteur Institute of Iran. Cancer cells were cultured in RPMI 1640 medium with foetal bovine serum. Then MCF7 cells were incubated with GNP‐PEG‐Paclitaxel with different concentrations of 43.125, 86.25, 172.5, 345 and 690 μM for 4 h. The selection of the concentrations for modified nanoparticles for “cell culture and cancer cell survival rate Study” was based on the results of previous experiments aimed at identifying the optimal drug concentration to be loaded on GNPs under different conditions. The optimisation studies revealed that a concentration of 690 μM exhibited the highest drug loading efficiency. Based on this finding, a sample with a concentration of 690 μM was prepared for cell culture testing and four additional concentrations were obtained by serial dilution from the optimised concentration. After the incubation period, the nanoparticles were removed from each well and fresh fetal bovine serum culture medium was added to the wells and the cells were incubated for 24 h. Then, the viability of cancer cells was evaluated using the MTT assay using the tetrazolium method. In this way, the percentage of cell viability was calculated.

**FIGURE 10:**
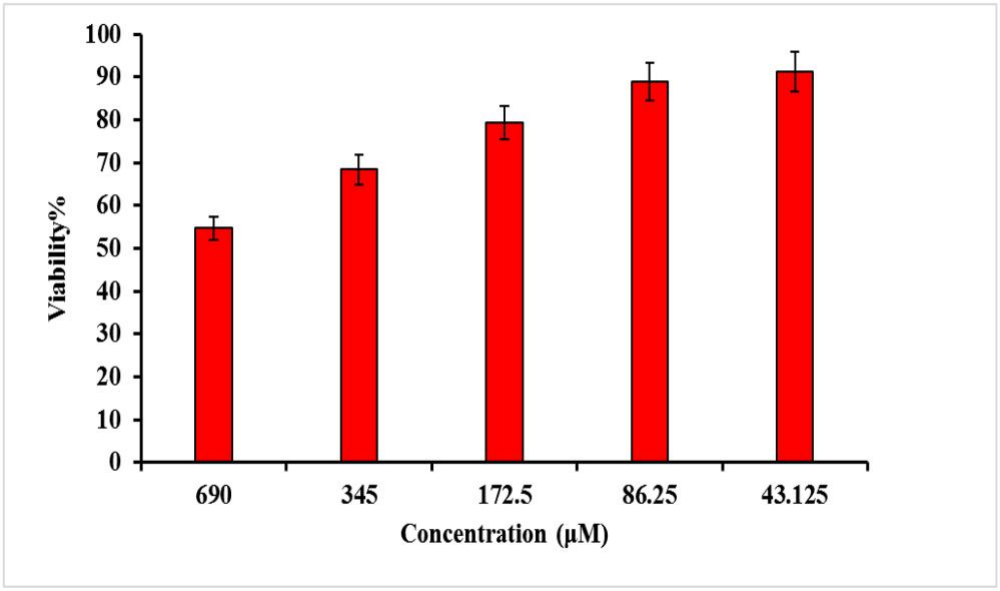
The viability percentage of MCF7 cells treated with various concentrations of GNP‐PEG‐Paclitaxel.

MCF7 cell viability incubated with different concentrations of GNP‐PEG‐Paclitaxel are presented in Figure 10. It was found that the survival percentage of MCF7 cells decreases to about 55% in the presence of the nanoparticles attached to paclitaxel at the maximum concentration measured (690 μM). In other words, this rate of cell death (45%) was significant (*p* < 0.05). Indeed, it is possible that free drug at certain concentrations may yield comparable or even superior results compared to the drug conjugated to nanoparticles. However, the primary objective of this study is to investigate the utilisation of an appropriate carrier for the paclitaxel drug in an aqueous physiological environment in order to prevent the accumulation of the organic drug and to enable multi‐modal cancer treatment by means of radiation therapy, sonodynamic therapy, and photothermal therapy in the presence of these nanoparticles. This is because, as numerous studies in the field of GNPs have shown, these particles in the presence of radiation, ultrasound, and laser result in vastly different outcomes compared to free drugs [3].

## 6 CONCLUSION

With the development of nanotechnology, different nanoparticles in different structures, shapes and composites have been able to provide good potential for cancer treatment. Compared to other nanoparticles, GNPs have many advantages in this field. Coating the surface of GNPs with a suitable and biocompatible material can make it a suitable carrier for anticancer drugs in the treatment of cancer cells. Paclitaxel is a first‐line chemotherapy agent for solid tumours, but its use is hampered by poor solubility, toxicity, and the emergence of resistance during treatment. In this research, it was tried to synthesise and evaluate GNPs coated with biocompatible polymers of PEG as paclitaxel drug carriers and the factors affecting the loading efficiency were examined. In general, in the tested experiments, it was found that PEG has a more effective role in the loading process of paclitaxel and GNPs have a limited loading capacity of paclitaxel on the nanostructure. In addition, the effect of incubation time and pH on the loading efficiency of paclitaxel on pegylated GNPs was investigated. It was found that at the time of 24 h of incubation and acidic environment, the loading capacity reached its maximum. Also, using the synthesised nanostructure in the treatment application of breast cancer cells was studied. The survival percentage of MCF7 cells in the presence of the paclitaxel‐bound nanoparticles decreases to about 55% at the maximum measured concentration (690 μM). In other words, this rate of cell death (45%) was significant (*p* < 0.05).

## AUTHOR CONTRIBUTIONS

Afrooz Kharazmi: Methodology, Writing—original draft. Neda Attaran: Conceptualisation, Data curation, Investigation, Project administration, Supervision, Writing—original draft, Writing—review & editing.

## CONFLICT OF INTEREST STATEMENT

The authors declared that they have no conflicts of interest to this work.

## Data Availability

Data sharing not applicable to this article as no datasets were generated or analysed during the current study.
